# Supplementary material for: A serological assay to detect SARS-CoV-2 antibodies in at-home collected finger-prick dried blood spots
Source: Sci Rep. 2020 Nov 19;10:20188. doi: 10.1038/s41598-020-76913-6 (PMC7678827; doi:10.1038/s41598-020-76913-6)
Supplement: Supplementary file 1 — Supplementary Information. [file 41598_2020_76913_MOESM1_ESM.pdf]

A serological assay to detect SARS-CoV-2 antibodies in at-home collected finger-prick dried blood spots

Donna Grace Karp[1],
Kenneth Danh[1],
Noemi Fonseca[1],
David Seftel[1],
Peter V. Robinson[1]*,
Cheng-ting Tsai[1]*
*Corresponding authors:
peter@enablebiosciences.com,
jasontsai@enablebiosciences.com

Affiliations:
1. Enable Biosciences Inc, South San Francisco, CA

SUPPLEMENTARY INFORMATION

S1. Investigation of COVID-19 dried blood spot specimen using non SARS-CoV-2 conjugates. Dried blood spot specimen may contain substantial cell debris that could potentially interfere the assay signals. It is thus of interest to affirm the SARS-CoV-2 antibody signals observed in COVID-19 patients in Figure 1 was indeed specific to SARS-CoV-2 antigen and not a result of matrix difference between COVID-19 patients and healthy controls. To that end, we re-tested dried blood spot specimens from 18 COVID-19 patients and 20 healthy controls using a non SARS-CoV-2 protein-DNA conjugates (*Borrelia burgdorferi* OspC protein). As expected, the signal distributions were statistically insignificant (P=0.83) between the two patient groups, affirming the specificity of the observed results in Figure 1.

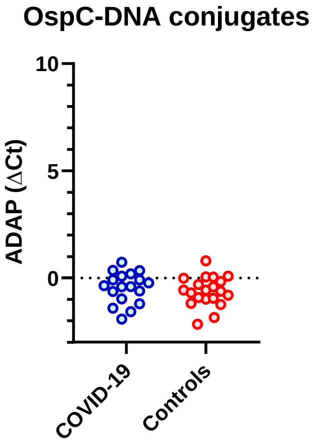

Figure S1. Analysis of COVID-19 (N=18) and healthy controls (N=20) dried blood spot specimen using *Borrelia burgdorferi* OspC protein-DNA conjugates. The COVID-19 patient signals were plotted in blue, while the healthy controls signals were in red. The assay signals were close to the baseline for both groups, and the difference were statistically insignificant.

S2. Investigation of COVID-19 dried blood spot specimen using protein G beads pull down. To further affirm the observed signals in Figure 1 from COVID-19 patients were indeed specific to immunoglobulins, we incubated dried blood spot eluent with protein G beads to remove the immunoglobulins. Then, we assayed the supernatant with the ADAP SARS-CoV-2-DNA conjugates. As expected, we observed a strong loss of signals from the COVID-19 patient group (P<0.001) while the healthy control signals remained close to the baseline after protein G beads pull down. Notably, one dried blood spot specimen from the COVID-19 group had a signal of 7.71 before pull down and 4.93 after pull down. While this represented a substantial loss of signals, the signals

were not completely removed. This might be explained by the fact that protein G beads had a strong capacity in removing immunoglobulins of IgG class but with lower capacity for IgM, IgA and other classes. This patient might have higher concentration of non-IgG class of SARS-CoV-2 antibodies. Taken together, the pull down experiment further affirmed the specificity of observed signals in Figure 1.

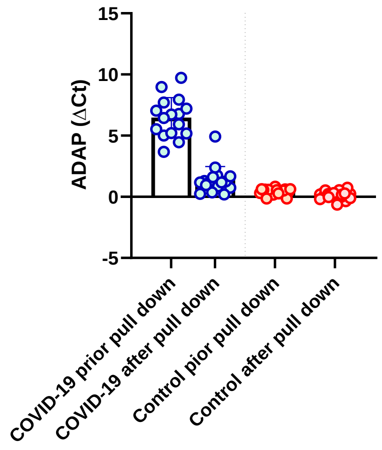

Figure S2. Analysis of dried blood spot specimen using ADAP SARS-CoV-2 DNA conjugates before and after protein G beads pull down. The COVID-19 samples (N=16) were plotted in blue, while the healthy controls (N=20) were plotted in red. The signal distribution for COVID-19 samples before and after pull down reached statistical significance (P<0.001).

S3. Analysis of ADAP SARS-COV-2 antibody assay signals based on collection distance.

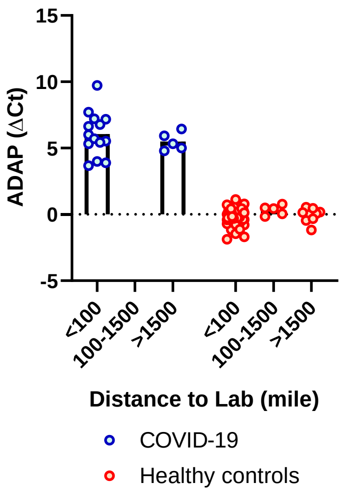

Figure S3. Finger-prick dried blood spot signals by distance to the testing lab. The

self-collected finger-prick samples were grouped into short distance (<100 miles), moderate distance (100-1500 miles) and long distance (>1500 miles) based on the geographical distance between the donor's resident city and South San Francisco (the site of the testing lab).

S4. Simulated summer temperature cycling conditions per FDA EUA template for home specimen collection molecular diagnostic template.

Temperature	Cycle Period	Cycle Period Hours	Total Time Hours
40°C	1	8	8
22°C	2	4	12
40°C	3	2	14
30°C	4	36	50
40°C	5	6	56

Table S4. Simulated summer temperature cycling conditions.

S5. Simulated winter temperature cycling conditions per FDA EUA template for home specimen collection molecular diagnostic template.

Temperature	Cycle Period	Cycle Period Hours	Total Time Hours
-20°C	1	8	8
22°C	2	4	12
-20°C	3	2	14
4°C	4	36	50
-20°C	5	6	56

Table S5. Simulated winter temperature cycling conditions.

S6. Evaluation of dried blood spot eluent stability. To investigate the dried blood spot eluent stability, we analyzed the fresh eluent and stored the eluent in -80°C after 1 week. The results showed that there were no statistical significant difference before and after the storage for both a COVID-19 specimen and a control specimen.

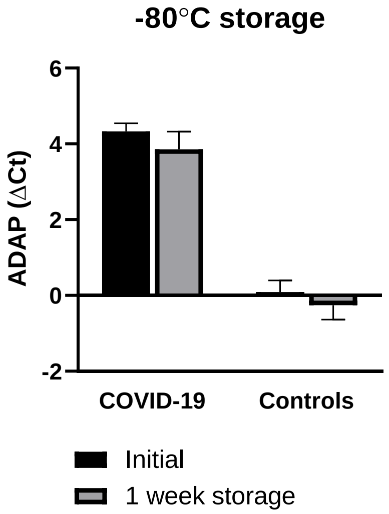

Figure S6. Analysis of dried blood spot eluent stability in -80°C. Five replicates of dried blood spots harboring low quantity of COVID-19 antibodies were eluted and then analyzed with SARS-CoV-2 DNA conjugates before (black) and after 1 week of storage (grey) in -80°C. The same were conducted for five replicates of dried blood spot not containing SARS-CoV-2 antibodies. The distribution of signals before and after cold storage for both COVID-19 and controls didn't reach statistical significance.

S7. Multiplex detection of dried blood spot eluent for S1 and N protein antibodies.

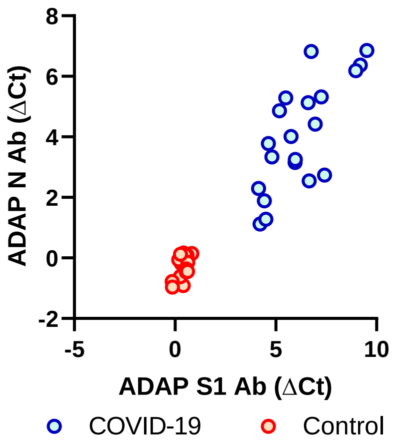

Figure S7. Multiplex analysis of SARS-CoV-2 antibodies in dried blood spot eluent. Dried blood spot eluents from COVID-19 patients (n=20, blue) and healthy controls (n=20, red) were analyzed by ADAP assay using S1 and N protein-DNA conjugates. The S1 protein antibody signals were plotted in the x-axis and the N protein antibody signals in the y-axis.
